# Characterisation of bird cherry‐oat aphid (*Rhopalosiphum padi* L.) behaviour and aphid host preference in relation to partially resistant and susceptible wheat landraces

**DOI:** 10.1111/aab.12616

**Published:** 2020-07-15

**Authors:** Beant Singh, Amma Simon, Kirstie Halsey, Smita Kurup, Suzanne Clark, Gudbjorg Inga Aradottir

**Affiliations:** ^1^ Punjab Agricultural University (PAU) Ludhiana India; ^2^ Rothamsted Research Harpenden UK; ^3^ School of Biosciences University of Nottingham Nottingham UK

**Keywords:** aphid, EPG, insect behaviour, resistance, *Rhopalosiphum padi*, wheat

## Abstract

The bird cherry‐oat aphid (*Rhopalosiphum padi* L.) is a major pest of wheat (*Triticum aestivum* L.) and can cause up to 30% yield losses. Heritable plant resistance to aphids is both an economically and ecologically sound method for managing aphids. Here we report how the behaviour and performance of *R. padi* differs on two resistant, one susceptible wheat landrace and a susceptible elite wheat variety. Feeding behaviour differed among the genotypes, with aphids on resistant lines spending longer in the pathway phase and less time phloem feeding. These behaviours suggest that both inter‐ and intracellular factors encountered during pathway and phloem feeding phases could be linked to the observed aphid resistance. Locomotion and antennal positioning choice tests also revealed a clear preference for susceptible lines. Although feeding studies revealed differences in the first probe indicating that the resistance factors might also be located in the peripheral layers of the plant tissue, scanning electron microscopy revealed no difference in trichrome length and density on the surface of leaves. Aphids are phloem feeders and limiting the nutrient uptake by the aphids may negatively affect their growth and development as shown here in lower weight and survival of nymphs on resistant genotypes and decreased reproductive potential, with lowest mean numbers of nymphs produced by aphids on W064 (54.8) compared to Solstice (71.9). The results indicate that resistant lines markedly alter the behaviour, reproduction and development potential of *R. padi* and possess both antixenosis and antibiosis type of resistance.

## Graphical abstract

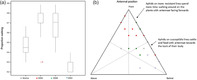

Aphid behaviour is dependent on the suitability of the host plant variety, where aphids on susceptible varieties settle more quickly and feed better than aphids on more resistant varieties which move about more and take longer to probe the plant. This results in reduced development and survival of aphids feeding on resistant lines as well as a reduction in nymph production.

## 1 INTRODUCTION

Wheat (*Triticum aestivum* L.) is one of the most important food crops in the world (Ortiz et al., 2008). Many insect pests have been reported to infest wheat worldwide. While most of these insects cause insignificant damage, others cause serious yield reduction across international borders (Miller & Pike, 2002). Of a number of aphid species which attack wheat crops, *Rhopalosiphum padi* L. is considered a major pest. It can cause up to 20–30% yield losses in cereal crops (Voss, Kieckhefer, Fuller, McLeod, & Beck, 1997). Aphids are phloem feeders and secrete honey dew onto the plant on which black sooty mould grows. This saprophytic fungus reduces the photosynthetic efficiency of plants (Rabbinge, Drees, Van der Graaf, Verberne, & Wesselo, 1981). Apart from direct damage and yield loss, *R. padi* can also vector plant viruses via the saliva (Rochow & Eastop, 1966). Currently, insecticides are applied with the aim to control aphids (Tanguy and Dedryver, 2009). However, insecticide resistance has been reported in aphids against major classes of insecticides (Bass et al., 2014; Foster et al., 2014). This, coupled with restrictions on the use of some pesticides in major wheat producing countries, has focused global research efforts to find alternative modes of controlling aphids (Loxdale, 2008; Sparks, 2013). Heritable plant resistance is an economically sound and ecologically safe method for managing aphids and sustainability of wheat production (Smith, 2005). With the threat of insecticide resistance in cereal aphids and the impending neonicotinoid ban coming into force in Europe, it is important to increase efforts to identify resistance in wheat to cereal aphids. Resistance to cereal aphids has been reported from a number of sources, such as *Triticum monococcum* L. (Greenslade et al., 2016), triticale (Hesler & Tharp, 2005), triticale‐derived germplasm (Crespo‐Herrera, Smith, Singh, & Åhman, 2013) and more recently from commercial cultivars grown in the United States (Girvin, Whitworth, Aguirre Rojas, & Smith, 2017).

Aphids are thought to assess internal plant chemistry by briefly puncturing the plant epidermal cells to accept or reject a host plant (Harris, 1977; Prado & Tjallingii, 1997). Stylets follow a largely intercellular path until they reach sieve elements, with phloem feeding being the ultimate step in successful host plant selection. Aphid probing behaviour depends on many plant resistance factors including barriers to stylet penetration in materials between plant cells, a lack of essential aphid nutrients in phloem components, or the presence of detrimental secondary compounds in phloem (Dixon, 1998). Aphid probing behaviour can be studied using the electrical penetration graph (EPG) technique which can provide information related to plant suitability to aphids, helping to understand the factors providing aphid resistance (Tjallingii, 2006).

The feeding behaviour of *R. padi* has previously been studied using EPG on wild relatives of wheat, *T. monococcum*, which showed that partial resistance was related to higher number and duration of salivation events without subsequent phloem feeding (Greenslade et al., 2016). Lower aphid growth rate and longer time to attain a committed phloem ingestion have been reported to be associated with wheat having higher levels of hydroxamic acid (Givovich & Niemeyer, 1994) although some studies have not been able to confirm that link (Pereira et al., 2017). Differences in cell anatomy have also been reported to be associated with insect pest resistance (Thimmaih, Panchal, Kadapa, & Nalini Parbhakar, 1993). Transmission electron microscopy suggests that the thick‐walled sclerenchyma cells around the vascular bundle play a role in southern chinch bug resistance in St. Augustinegrass, possibly by reducing stylet penetration to the vascular tissue (Rangasamy, Rathinasabapathi, McAuslane, Cherry, & Nagata, 2009).

Recently, partial resistance to *R. padi* has also been identified in some of the Watkins landrace wheat collection accessions in the United Kingdom (Aradottir, Martin, Clark, Pickett, & Smart, 2016). The Watkins collection was assembled in the 1920s, representing a selection of landrace wheats from 32 countries around the world. The collection totals 1,291 lines, with a core collection comprising 119 lines capturing the majority of the genetic diversity (Wingen et al., 2014). New genes for rust and root‐lesion nematode resistance have been already identified in the Watkins collection (Bansal et al., 2011; Dyck, 1994; Thompson & Seymour, 2011). Thus, detailed studies on understanding the post‐alighting behaviour on Watkins wheat expressing antibiosis resistance may provide information useful to incorporate resistance genes into improved cereal crop cultivars.

## 2 MATERIALS AND METHODS

Three types of experiments (EPG, locomotory and antennal positioning bioassay, reproduction and development studies) were conducted to ascertain the settling and feeding behaviour of *R. padi* on selected lines from the Watkins wheat collection.

### 2.1 Plants, aphids and environmental conditions

Seeds of partially resistant wheat lines W068 and W064, as well as the susceptible line W591 were obtained from the Germplasm Unit at the John Innes Centre, United Kingdom, and tested along with the hexaploid wheat *T. aestivum* var. Solstice which is known to be susceptible to *R. padi*. The seeds of each genotype were planted in Rothamsted Prescribed Mix (supplied by Petersfield Products, Leicestershire, UK) which is composed of 75% medium grade (L&P) peat, 12% screened sterilised loam, 3% medium grade vermiculite and 10% grit (5 mm screened, lime free). A mixed culture of *R. padi*, collected from wheat fields near Rothamsted Research, Harpenden, Hertfordshire, UK, were reared in independent ventilated Perspex cages on susceptible “Saffron” barley (*Hordeum vulgare* L.).

Environmental conditions for plants, insect and experiments were all identical: 20°C temperature, 60–70% humidity and a photoperiod of 16:8 hr (L:D), with daily watering. Plants were tested at developmental stage 10, as described by Zadoks, Chang, and Konzak (1974).

### 2.2 Electrical penetration graph experiment

Feeding behaviour of *R. padi* was studied by EPG using the methodology described by Tjallingii (1988, 2000). A gold wire (18 μm) electrode was attached to the dorsum of each adult apterous aphid with the aid of a specially adapted suction pump and water‐based adhesive containing silver paint. The paint was also used to connect the gold wire to a piece of 2.5–3 cm copper wire, which was connected in turn to a brass pin via solder. This apparatus was then connected to an 8‐channel “Giga‐8” DC amplifier of 1 GΩ input resistance (EPG‐systems, Wageningen, the Netherlands) housed in a grounded Faraday cage. The first leaf of a wheat plant was secured to the base of an upside down 100 ml Pyrex® beaker using two pieces of clear plastic tape (2.5 × 0.5 cm) on the two edges where the leaf blade met the circumference to restrict plant movements, but without applying pressure to the leaf blade itself. A Petri dish filled with water was placed under each pot and the plant watered so that the soil was saturated to ensure good electrical conductivity throughout the duration of the experiment. An electrode was then placed in the soil, the aphid put on the plant and an 8‐hr EPG recording commenced using Stylet+data acquisition software (EPG‐systems, Wageningen, the Netherlands). All recordings were made between 11.00 a.m. and 8.00 p.m., with room temperature maintained at 20°C and a constant light level provided by three 80 W fluorescent lights. Positions of the plants and probe wires were randomised for each run. Two replicates of each of four lines were run per day. EPG waveform recordings were interpreted using the Stylet+ analysis software, annotated and imported into version 10.6 m of the EPG analysis Microsoft Excel macro (available from Dr Schliephake via EPG‐systems, Wageningen, the Netherlands) to calculate feeding behaviour parameters from the waveforms. Aphid waveforms were placed into the following categories: non‐probing (Np), stylet pathway phase containing waveforms A, B and C (C), phloem sieve element salivation (E1), phloem sieve element ingestion (E2), derailed stylet mechanic/penetration difficulties (F) and xylem drinking (G) (Pettersson, Tjallingii, & Hardie, 2007; Tjallingii, 1988, 2000). Prior to recordings, plants and aphids were transferred to the laboratory and allowed to acclimatise for approximately 1 hr. Twenty replicates were performed for each genotype, but only replicates where feeding behaviour was observed within the first hour and for at least 30 min within the last hour of recording were included in the analysis, leading to 11–18 qualifying replicates per line (Table 1).

**TABLE 1:** List of electrical penetration graph variables

Variables	Solstice	W064	W068	W591	*p*	Transformations
Sample size of qualifying replicates	14	18	11	15		
*Probing (tissue penetration)*						
Time to first probe	0.863^a^	2.209^b^	2.384^b^	1.269^a^	<0.001	Log
Duration of first probe	2.398^b^	3.248^ab^	3.45^a^	2.843^ab^	0.057	Log
Number of probes	2.512	2.481	2.857	2.535	0.817	Sqrt
Number of brief probes	0.995	0.881	1.441	1.035	0.516	Sqrt
Average probe length	41.2	46.55	52.86	42.73	0.357	Sqrt
Total time probing	12,698	14,497	18,236	15,905	0.497	None
*Pathway*						
Number of pathway phases (C)	26.05	39.03	37.35	23.76	0.052	None
Average time of the pathway (C)	16.1^ab^	13.71^b^	15.17^ab^	17.4^a^	0.01	Sqrt
Time to first potential drop (pd) (from start of first probe)	1.837	1.735	2.056	1.455	0.159	Log
Number of potential drops (pd) to first phloem event (E)	1.064^a^	0.995^ab^	0.709^ab^	0.621^b^	0.03	Log
*Salivation*						
Number of single salivation events (sgE1)	2.146^ab^	2.505^ab^	2.963^a^	1.782^b^	0.044	Sqrt
Average single salivation events (sgE1)	2.081	2.124	1.971	1.904	0.342	Log
Number of salivation events (E1)	11.45	12.45	15.87	15.04	0.543	None
Average salivation events (E1)	2.254	2.2	2.248	2.078	0.523	Log
*Phloem feeding*						
Number of phloem feeding events (E2)	0.675^ab^	0.620^b^	0.523^b^	0.883^a^	0.021	Log
Average phloem feeding events (E2)	3.45	3.296	3.067	3.151	0.39	Log
Total phloem feeding duration (E2)	13,336^a^	9,225^ab^	6,840^b^	14,963^a^	0.012	None
Maximum phloem feeding event (E2)	12,158^a^	7,940^ab^	5,051^b^	10,175^ab^	0.038	None
Number of sustained phloem feeding events (sE2)	1.415^ab^	1.078^b^	0.939^b^	1.501^a^	0.014	Sqrt
Time to first phloem feeding (E2)	71.21^b^	83.18^ab^	105.45^a^	61.39^b^	0.006	Sqrt
Time to first phloem feeding from first salivation (E1 to E2)	2.351^bc^	3.042^ac^	3.306^a^	1.991^b^	<0.001	Log
Time to first sustained phloem feeding (sE2)	8,989^b^	15,695^a^	17,565^a^	8,992^b^	0.003	None
*Xylem drinking and total feeding time*						
Number of xylem drinking (G)	0.5105	0.4262	0.5679	0.3895	0.488	Log
Average xylem drinking (G)	2.817	2.979	3.111	3.075	0.223	Log
Time to first xylem drinking (G)	3.188	3.201	2.966	3.461	0.184	Log
Sum of E1 and E2	16,203	12,059	9,726	16,465	0.042	None
Per cent total feeding time	56.26	41.87	33.77	57.17	0.042	None

*Note:* Total duration (in seconds), frequency and average duration (predicted means) from 8 hr of recording of *R. padi* feeding on Watkins wheat lines W064, W068, W0591 and *T. aestivum* var. Solstice. Letters indicating significant differences between the lines are based on adjusted confidence intervals which allow for all pairwise comparisons.

### 2.3 Locomotory and antennal positioning bioassays

These behaviour bioassays were conducted to test the hypothesis that aphids cannot find a suitable position to probe or penetrate the wheat tissue on resistant genotypes, whereas on susceptible genotypes, the aphid will settle down more quickly with the characteristic antennal position indicative of feeding behaviour. Choice studies were performed to assess aphid preference among the three Watkins lines (W591, W064, W068) and Solstice, as before. Prior to introduction, aphids were placed in a Petri dish and starved for approximately 1 hr. Following the pre‐treatment, a single adult apterous aphid was introduced in the centre of the leaf using a fine, wet camel hair brush. The aphids were placed on the first leaf of each genotype. At the end of each minute within a 10‐min period the aphid's behaviour was recorded. The behaviours were categorised as walking or still (locomotory) and antennae in front, above or behind the head (antennal positioning).

### 2.4 Aphid development and reproduction assay

Resistant and susceptible wheat lines were sown singly into pots of Rothamsted Prescribed Mix as in the behavioural bioassay previously described. There were 10 replicates of each genotype with the experiment set up as a randomised complete block design. Two adult alate aphids were placed within clip cages (2 cm diameter) and placed onto 7‐day‐old plants, as described by MacGillivray and Anderson (1957) and allowed to larviposit for 24 hr, when they were removed and the number of nymphs produced recorded. Neonate nymphs (<1 day old) were weighed using a Microbalance (Cahn 33; Scientific and Medical Products Ltd, Manchester, UK) and transferred back to a plant of the same genotype and left undisturbed for 7 days. After 7 days, the number of survivors were recorded and survivors re‐weighed to determine the mean relative growth rate (mRGR; Radford, 1967; Leather & Dixon, 1984),

$$\text{mRGR}=\left(\frac{\ln\left(\text{seven}\;\mathrm{day}\;\text{weight}\right)-\ln\left(\text{birth weight}\right)}{6}\right).$$

After re‐weighing, one of the nymphs was chosen at random and transferred back to their original plant. Aphids were then left undisturbed to develop and monitored daily until moulting into adult apterous aphids. The time taken to produce the first nymph (FD) and the number of nymphs produced over their lifetime (D) were recorded to calculate the intrinsic rate of increase. The constant 0.74 is an approximation of the proportion of the total fecundity produced by a female in the first D days of reproduction (Awmack & Leather, 2007).

$$r_{m}=0.74(\frac{\ln\left(\mathrm{FD}\right)}{\left(D\right)}.$$

### 2.5 Scanning electron microscopy

Leaf surface morphology was studied using scanning electron microscopy (SEM) to discern any noticeable differences of leaf surfaces. Seedlings of all four genotypes were grown to developmental stage 10 (Zadoks et al., 1974) and the first fully expanded leaves were cut into 5 mm sections using a scalpel. The leaf sections were mounted on an aluminium stub using a 50:50 mix of Tissue‐Tek OCT compound and colloidal graphite. The samples were rapidly frozen in liquid nitrogen then transferred to the GATAN Alto 2100 cryo prep chamber. They were etched at −95°C for 2 min to remove any ice contamination before being coated with a thin layer of gold. Samples were then transferred to the JEOL 6360 LV SEM and imaged using an accelerating voltage of 5 kV.

### 2.6 Light microscopy

Leaf samples (n = 5) were chemically fixed in 4% (wt/vol) paraformaldehyde and 2.5% (wt/vol) glutaraldehyde in 0.05M Sorenson's phosphate buffer pH 7.2. Samples were washed three times in 0.05M Sorenson's phosphate buffer, dehydrated in a graded ethanol series and infiltrated in increasing concentrations of LR White Resin (medium grade Agar, AGR1281). Samples were polymerised at 60°C for 16–20 hr in a nitrogen rich environment and semi‐thin sections (1 μm) cut using a Leica rotary microtome RM 2265 (Leica Biosystems, Milton Keynes, UK). Sections were collected on drops of distilled water on glass slides coated with poly‐l‐lysine and dried on a hot plate at 60°C. The sections were stained with 1% (wt/vol) Toluidine blue in 1% (wt/vol) sodium tetraborate buffer pH 9 for 1 min and rinsed in distilled water for 1 min. Toluidine blue was used to highlight general histological features. Images of tissues of different genotypes were acquired with a Zeiss Axiophot light microscope (Carl Zeiss Ltd., Cambridge, UK) equipped with a Q‐Imaging Retiga Exi Fast 1394 monochrome camera (QImaging, Surrey, BC, Canada) and Metamorph imaging software version 7.8.13 (Molecular Devices, LLC, Sunnyvale, CA).

### 2.7 Image analysis

Light and SEM images were analysed with the ImageJ version 1.48 software (National Institutes of Health) and the Fiji plugin. Sixty light microscopy images from four plants per line (n = 15) were used for counting cells in a 100‐μm wide transect and for measuring leaf thickness, size of vascular bundle, thickness of bundle sheath cells and size of the phloem. Cell number was determined for each tissue type (upper epidermis, palisade parenchyma, spongy parenchyma, lower epidermis and vascular bundle) and expressed as cell number per tissue type within a 100‐μm transect. Cell density was determined by dividing the number of cells in each tissue type by the area of this specific tissue type within the 100‐μm wide transect, and expressed as cell number per μm^2^.

### 2.8 Data analysis

First, the data were tested for conformity to assumptions of analysis of variance (ANOVA) as dictated by tests of normality and homogeneity of variance (Gomez & Gomez, 1984). Normality was assessed for all parameters using graphical analysis of residuals. Appropriate transformation was performed for data that did not follow a normal distribution. The variables with zeros required an offset to be added before taking logs; these were set at half the minimum non‐zero value recorded. The EPG recordings were analysed using a linear mixed model fitted using restricted maximum likelihood (REML). Hypothesis testing was carried out at the 5% significance level. The locomotory and antennal positioning data were analysed using a log‐linear model. Cell number and size of different regions of leaf tissues were first compared between Solstice and Watkins lines using a one‐way analysis of variance (ANOVA). All three Watkins genotypes were nested within “non‐Solstice” lines for comparison among themselves. All analyses were performed in Genstat (18th edition; VSN International, 2015).

## 3 RESULTS

### 3.1 EPG feeding behaviour

The statistical analyses of behavioural variables recorded through EPG revealed that for a number of variables *R. padi* fed more effectively on Solstice and W591 compared to W064 and W068 genotypes (Table 1). The lower number of replicates for W068 was because of the lack of feeding activity of less than 30 min by aphids in the last hour of recording.

#### 3.1.1 Probing phase

Statistically significant differences were recorded in the time to first probe in tested genotypes (*F* = 10.81; df = 3, 51.8; *p* < .001). It took approximately twice as long for aphids to probe the partially resistant lines (W064 and W068) for the first time compared to susceptible lines (lines W591 and Solstice). The average duration of first probe also seemed to be slightly longer (*F* = 2.67; df = 3, 51; *p* = .057) on W068 than on Solstice. However, no difference was found in number of probes, brief probes, average probe length or total probing time among different genotypes.

#### 3.1.2 Pathway phase and reaching the phloem

A difference was observed between the varieties in the number of pathway periods, when the aphid stylet is passing through the plant tissue on the way to the phloem (*F* = 2.75; df = 3, 50.4; *p* = .052). There was also difference in the average duration of pathway phase, with the longest pathway phase in W591 and the shortest in W064 (*F* = 4.23; df = 3, 48.5; *p* = .01). Fewer potential drops (stylet entry into a non‐target cell) were observed prior to first phloem feeding in W591 compared to Solstice (*F* = 3.03; df = 3, 46.8; *p* = .038). However, no difference was observed in time to the first potential drop within a probe.

#### 3.1.3 Salivation phase

There was no difference in how often and for how long the aphids salivated, whereas differences were observed in the number of times aphids salivated without ingesting phloem content (single salivation event) between the lines (*F* = 2.89; df = 3, 51; *p* = .044). These were highest in W068 and lowest in W591; however, the duration of this feeding behaviour did not differ.

#### 3.1.4 Phloem feeding and xylem drinking

The number of phloem feedings events were significantly fewer in W064 and W068 genotypes as compared to W591 (*F* = 3.56; df = 3, 48.8; *p* = .021). The total phloem feeding duration was greater in Solstice and W591 than in W068 (*F* = 4.02; df = 3, 52.1; *p* = .012) and the duration of maximum phloem feeding event (*F* = 3.01; df = 3, 52.4; *p* = .038) was longest in Solstice and shortest in W068. There was a difference in time to first phloem feeding, where the aphids took longest to establish phloem feeding on W068 (*F* = 4.68; df = 3, 50; *p* = .006), time to first sustained phloem feeding took almost twice as long in W064 and W068 as in W591 and Solstice (*F* = 5.27; df = 3, 50.9; *p* = .003) and first phloem feeding from first salivation event (*F* = 7.95; df = 3, 46.6; *p* < .001) was delayed for aphids feeding on W064 and W068 compared to W591. However, there was no difference in average duration of phloem feeding among different genotypes.

#### 3.1.5 Xylem drinking and total feeding time

No differences were observed in xylem drinking by *R. padi* on the lines. There was a difference in total time spent feeding (*F* = 2.93; df = 3, 51.2; *p* = .042) as well as the percentage of time spent feeding out of the recorded 8 hr was lowest on W068 (33.77%) and highest on W591 (57.17%).

### 3.2 Locomotory and antennal positioning bioassays

**FIGURE 1:**
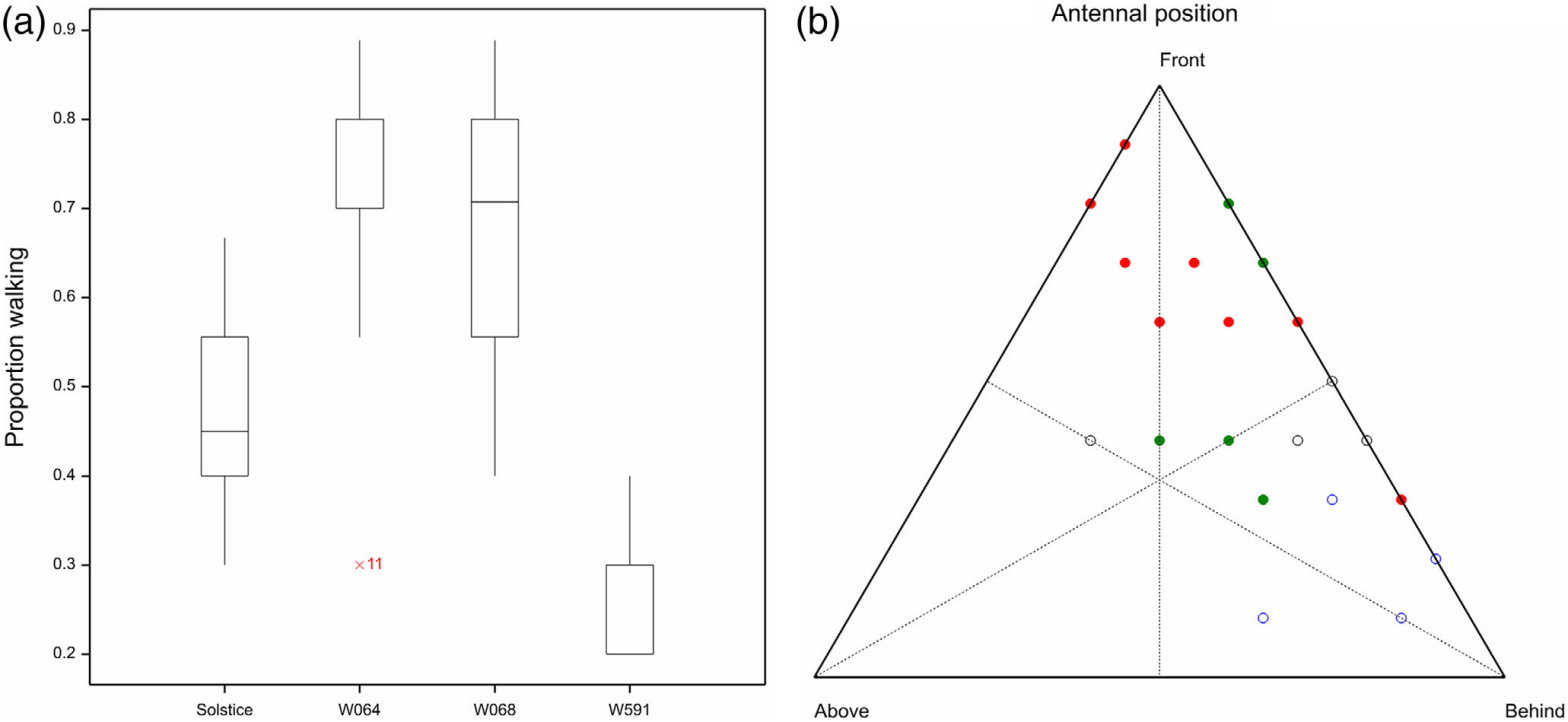
a) Boxplots for locomotory behaviour (% time walking) and b) ternary diagram for antennal behaviour by variety of Rhopalosiphum padi on Triticum aestivum var. Solstice (black open) and Watkins landraces W591 (blue open), W068 (green solid) and W064 (red solid)

There was a difference in locomotory behaviour (chi squared = 50.84; df = 3; *p* < .001) and antennal positioning (chi squared = 45.05; df = 6; *p* < .001) among different wheat genotypes (Figure 1). Aphids tended to move with antennae in front of their head on resistant Watkins lines (W064 and W068) and behind their head on W591 and Solstice.

### 3.3 Aphid development and reproduction assay

**FIGURE 2:**
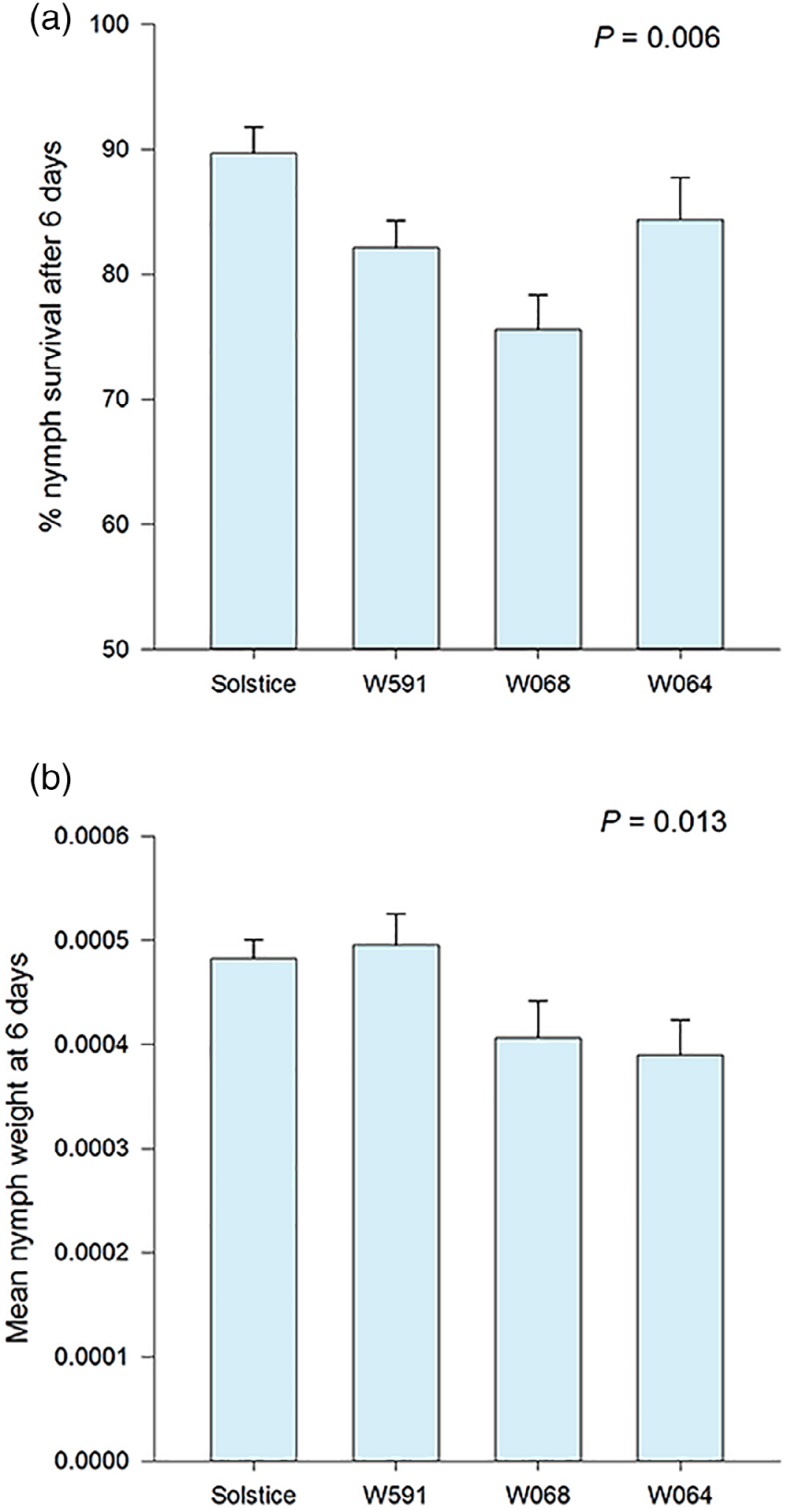
(a) Mean survival and (b) weight of *R. padi* 6 days after their release on *T. aestivum* var. Solstice and Watkins landraces W591, W068 and W064

**FIGURE 3:**
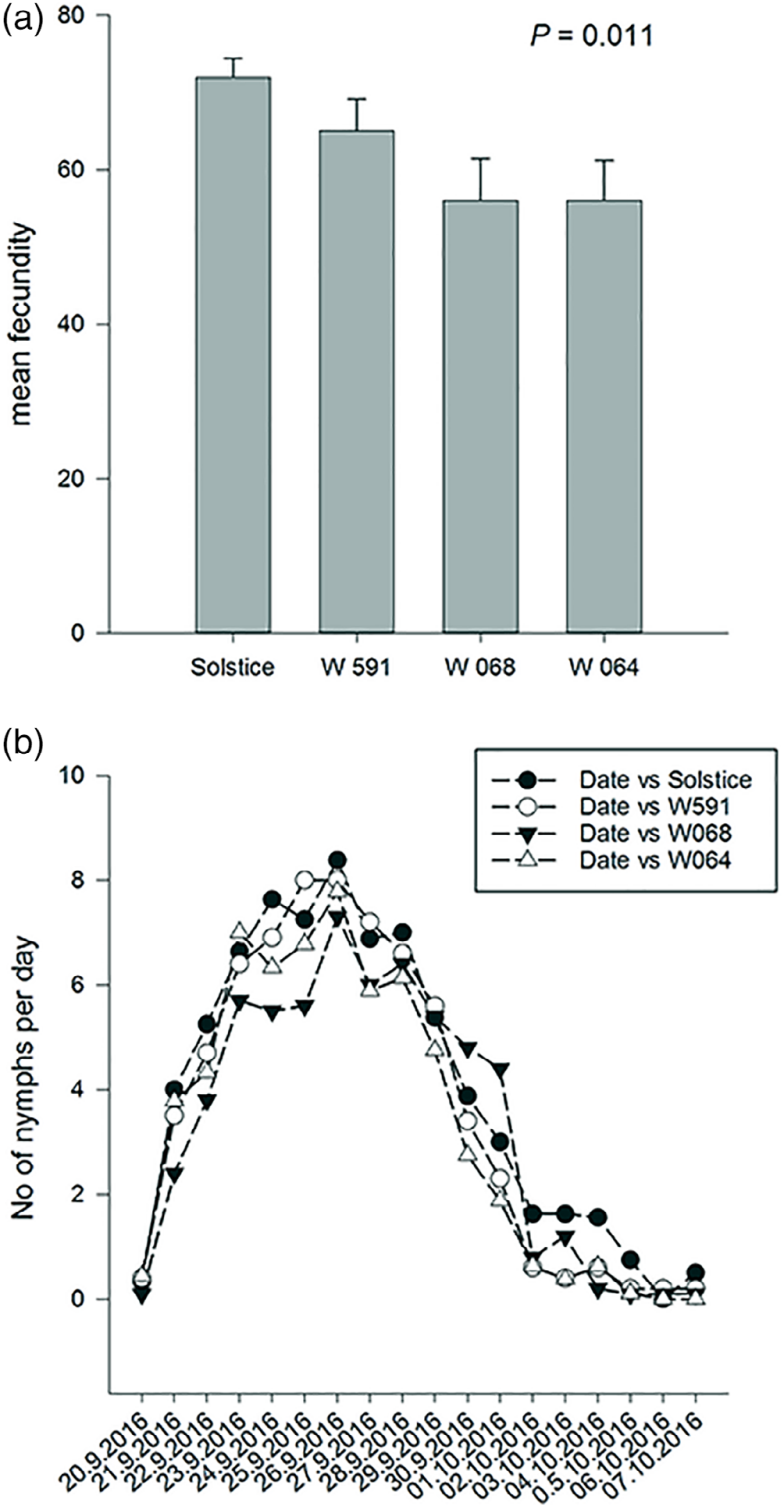
(a) Total and (b) relative daily fecundity of *R. padi* on *T. aestivum* var. Solstice and Watkins landraces W591, W068 and W064

There was no difference in weight or number of nymphs laid during the 24 hr after alate introduction to the plants (*p* > .05). However, the weight of 6‐day‐old nymphs varied among the cultivars (*F* = 4.36; df = 3, 25; *p* = .013). The average weight of a nymph was lower on W068 (386 mg) and W064 (395 mg) compared to Solstice (496 mg) and W591 (495 mg; SED = 41.2 mg). This was coupled with a difference in survival of 6‐day‐old nymphs which was lowest on W068 (76.8%) and highest on Solstice (90.3%; *F* = 5.38; df = 3, 25; *p* = .005; Figure 2). Aphids started laying nymphs on average six to 7 days from birth and total fecundity differed with aphids on Solstice laying the highest mean number of nymphs (71.9) and aphids on W064 the lowest (54.8; SED = 5.26; *F* = 4.58; df = 3, 24; *p* = .011) (Figure 3).

### 3.4 SEM and light microscopy

**FIGURE 4:**
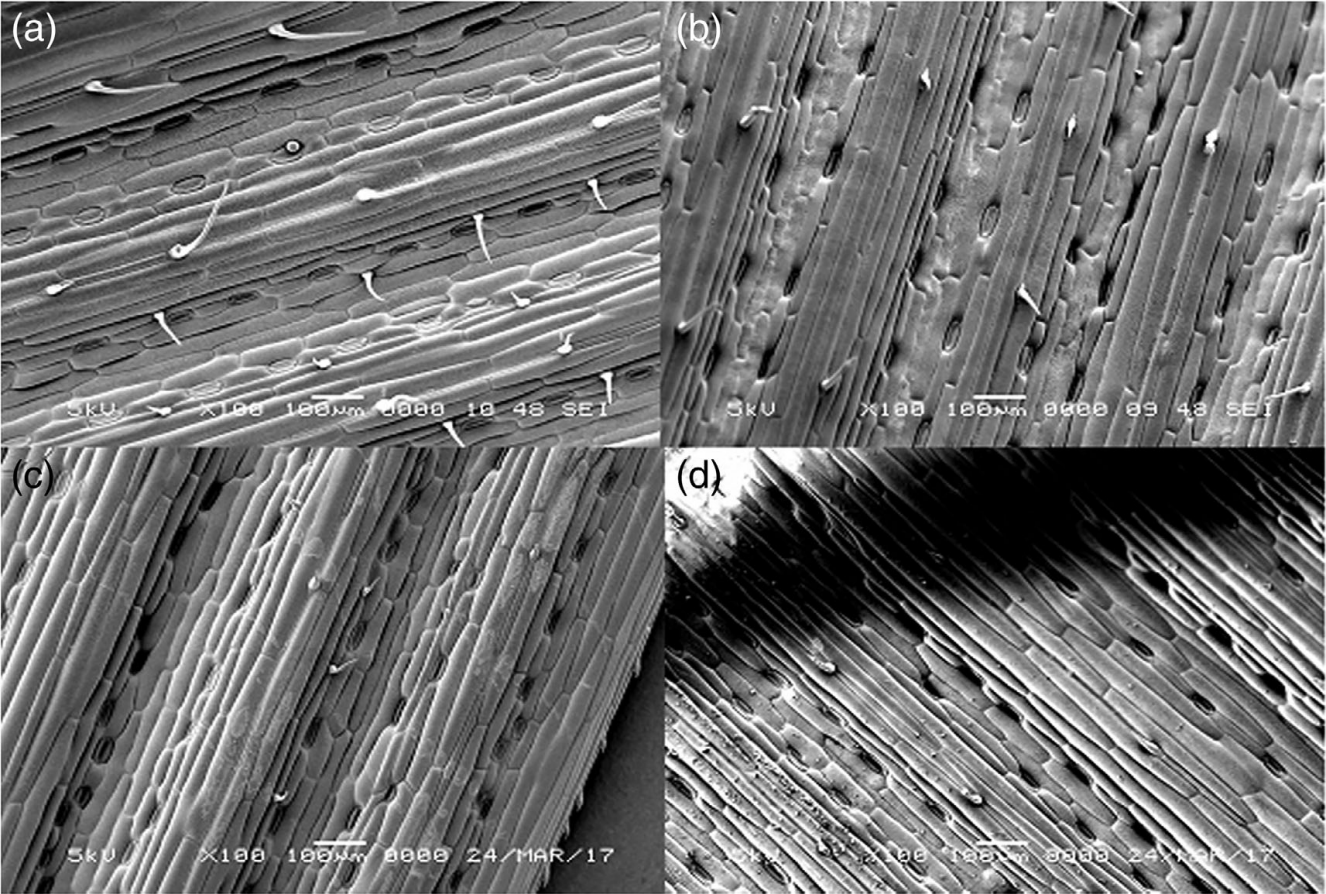
Scanning electron micrographs of leaf surfaces of four different wheat plants: (a) Solstice, (b) W064, (c) W068, (d) W591

There were no obvious differences in overall leaf morphology among the lines except for the presence of numerous trichomes on the upper surface of Solstice (Figure 4a), which appeared to be more numerous and longer than those on Watkins lines (Figure 4b–d).

**FIGURE 5:**
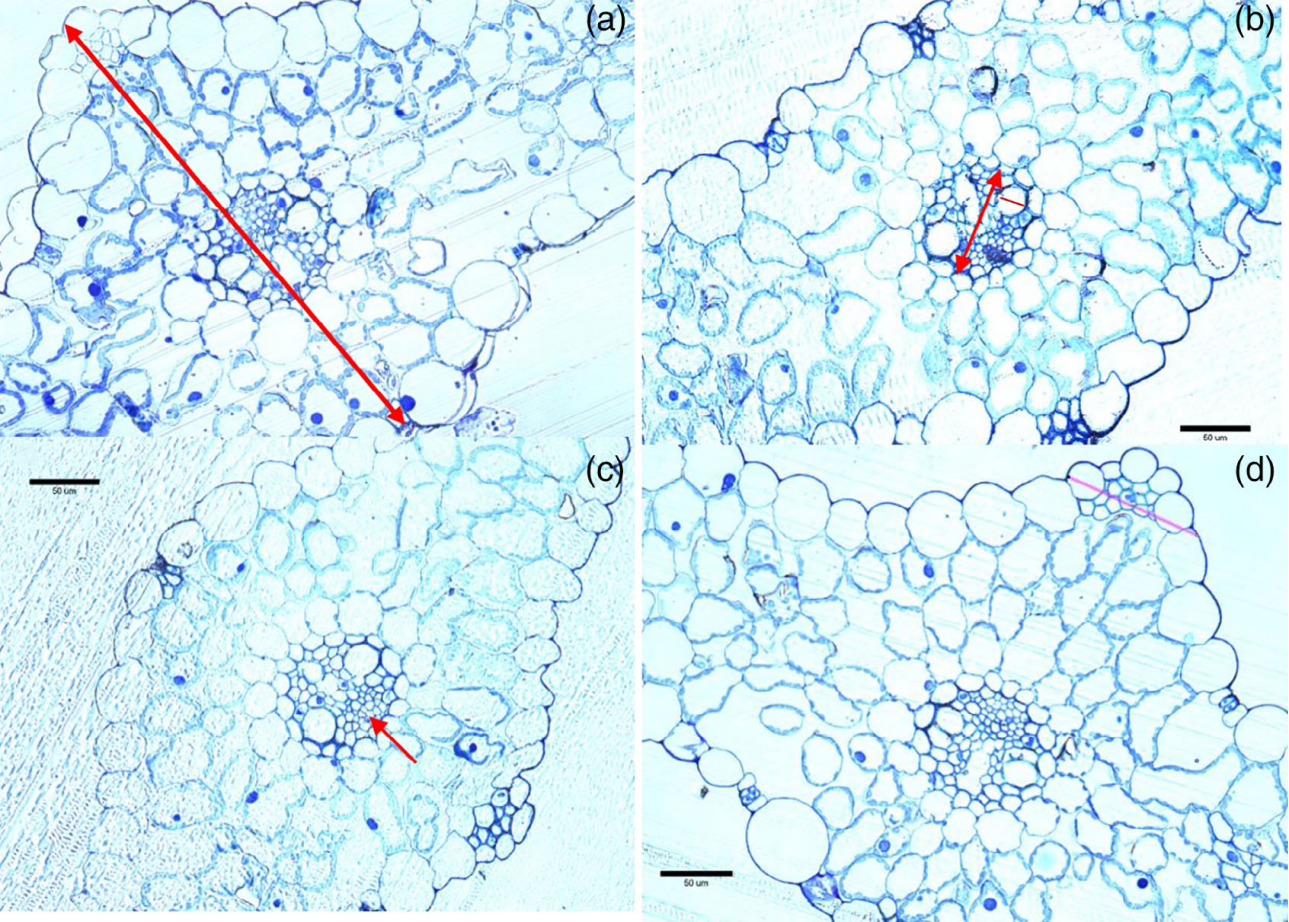
Morphology of leaf surfaces of four different wheat plants: (a) Solstice, (b) W064, (c) W068, (d) W591

Leaf thickness differed between the lines. The leaf was thinner in the modern hexaploid Solstice (321.53 ± 37.38 μm) than in the Watkins lines (366.1 ± 31.26 μm; *p* < .001; Figure 5a), whereas the leaf thickness (Figure S1, Supporting Information) did not differ significantly among Watkins leaves (W064 = 357.71 ± 13.50 μm, W068 = 366.74 ± 63.81 μm, W591 = 373.82 ± 16.46 μm; *p* = .52; Figure 5b–d). The size of the vascular bundle (Figure S1) did not differ significantly between Solstice (7705.73 ± 670.06 μm^2^; Figure 5a) and Watkins lines (7833.41 ± 787.78 μm^2^; *p* = .591; Figure 5b–d); however, vascular bundle size differed among the Watkins lines (*p* = .023). The vascular bundle of W591 (8251.73 ± 1077.82 μm^2^) was largest followed by W068 (7818.87 ± 504.76 μm^2^) and W064 (7429.62 ± 780.78 μm^2^). The size of the bundle sheath cells (Figure 5c) of Solstice (2664.76 ± 259.71 μm^2^; Figure 5a) were much smaller than in Watkins lines (2931.75 ± 276.66 μm^2^; *p* = .003; Figure 5b–d), whereas no difference was observed among Watkins lines (W064 = 2893.03 ± 361.18 μm^2^, W068 = 2855.47 ± 147.82 μm^2^, W591 = 3046.75 ± 320.98 μm^2^; *p* = .161; Figure 5b–d). The size of the phloem (Figure S1) did not differ between the lines. The number of mesophyll cells in 100 μm transect area (Figure S2) were significantly lower in Solstice (28.60 ± 2.07; Figure 5a) compared to Watkins lines (34.13 ± 0.84; *p* < .001; Figure 5b–d), whereas there was no difference among Watkins lines (W064 = 34.60 ± 0.54, W068 = 33.2 ± 1.09, W591 = 34.6 ± 0.89; *p* = 3.63; Figure 5b–d).

## 4 DISCUSSION

Plant resistance is one of the most effective methods for controlling insect pests (Smith, 2005; Smith & Boyko, 2007). Differential resistance to Russian wheat aphid has been demonstrated in wheat and barley (Khan et al., 2015) with resistant varieties regularly used in affected areas. Greenslade et al. (2016) found differential aphid resistance to *R. padi* in *T. monococcum* and reported that aphid resistance was closely linked to the feeding behaviour of sucking insect pests. Hence, monitoring the feeding process can reveal the behavioural mechanism of plant resistance. The use of EPG continues to be a valuable tool to determine causal factors associated with feeding behaviour of aphids. In the present study, resistant factors in W064 and W068 contributed to aphids spending more time in the pathway phase and less time feeding on phloem sap than aphids feeding on susceptible W591 and Solstice. Alvarez et al. (2006) reported that resistance factors in the epidermis and mesophyll may be indicated by a large number of test probes and an increased time in pathway phase. These behaviours could suggest that both inter‐ and intracellular factors encountered during the pathway and phloem feeding phases are linked to the observed aphid resistance in W064 and W068. A smaller number of mesophyll cells, indicating large intercellular space, thinner leaves and lower thickness of guard cells of vascular bundle could be possible reasons for the susceptibility of the susceptible hexaploid *T. aestivum* var. Solstice in the present investigation. The same morphological features were not observed for W591 however, which was more like the other Watkins lines.

Electrical penetration graph recordings revealed differential probing behaviour in *R. padi*. Similar results have been reported in tetraploid switchgrass against *Schizaphis graminum* (Koch et al., 2015). Locomotory and antennal positioning choice studies for *R. padi* also supported these results and revealed a clear preference for plants of Solstice and W591 relative to the other two lines from the Watkins wheat collections. This suggests that the resistant Watkins lines are repulsive to the aphids and that they were more satisfied with the surface of the susceptible wheat leaf for probing with their stylets. The present studies can therefore help breeders to select aphid resistance germplasm by monitoring these behaviour responses. EPG studies showed that aphids probed more quickly on Solstice and W591 compared to other genotypes which suggests that resistance factors might also be located in the peripheral layers of the plant tissue. This indicates that aphids encounter some physical barriers along the peripheral tissues. However, superficial plant characteristics in present investigation (Figure 4) did not appear to play an important role in influencing the settling and feeding behaviour of the aphids on these lines. Scanning electron microscopy (SEM) showed differences in trichrome length on the upper side of leaves, but the replication was insufficient for analysis, and further work is required to explain whether the barriers on the leaf surface are of a structural or chemical nature. In addition to the barriers to initial probing, the ability to phloem feed is crucial to aphids. Here the aphids spent ~2‐fold more time phloem feeding and had a higher number of sustained phloem feeding events (<10 min) on the susceptible Solstice and W591 compared to the resistant genotypes. The percentage of time the insect spends in sieve elements is a corrected index used to determine the acceptability of phloem (Dowd & Johnson, 2009; Tjallingii, 2000).

Differences in phloem acceptability likely explain the significant increase in the number of pathway phases in W064 and W068. Because each phase is mutually exclusive, *R. padi* feeding on the susceptible W591 and Solstice would have less time available for other phases, such as pathway, as more time was spent in the sieve element phase (Van Helden & Tjallingii, 2000). However, aphids feeding on resistant plants may continue probing, searching for a suitable feeding site, thereby leading to a greater number of pathway phases. In the experimental setting aphids are tethered to the plant and do not have the option of looking for an alternative. Phloem‐based mechanisms of resistance to aphids have previously been reported, including resistance in melon genotypes (*Cucumis melo* L.) to the cotton melon aphid, *Aphis gossypii* (Garzo, Soria, Gómez‐Guillamón, & Fereres, 2002). Such resistance could be because of physical (i.e., difficulty overcoming phloem wound response) or chemical mechanisms (i.e., deterrent compounds in sieve tubes; Greenslade et al., 2016; Tjallingii, 2006; Le Roux et al., 2008). Aphids are phloem feeders and limiting the nutrient uptake by the aphids will negatively affect their growth and development. Indeed, it forms the basis of antibiosis type of resistance which often leads to a strong deterrent effect resulting in a weakened physiological condition (Smith, 2005). Relatively lower weight of 6‐day‐old nymphs on resistant genotypes (W064 and W068) in present studies also support this fact. It not only affects the growth and development of aphids but also decreases their reproductive potential as less progeny were produced and a lower survival (%) of nymphs shown on resistant W064 and W068. Metabolic phenotyping of *T. monococcum* revealed that aphid resistant genotypes have lower levels of primary metabolites including total carbohydrates (Greenslade et al., 2016). However, asparagine and octopamine, threonine, glutamine, succinate, trehalose, glycerol, guanosine and choline increased in response to aphid infestation in susceptible genotypes. Further studies are required on the Watkins accessions used in the present study to assess the role of plant chemistry in resistance.

This research provides the first detailed documentation on the feeding behaviour of aphids on Watkins wheat collections. The results indicate that resistant lines W064 and W068 markedly altered the behaviour of *R. padi* and that W064 and W068 may possess both antixenosis and antibiosis resistance to *R. padi*. Combinations of resistance categories are often reported, including many examples of antibiosis and antixenosis together (Castro, Martin, & Martin, 2006; Garzo et al., 2002; Hawley, Peairs, & Randolph, 2003). The combination of multiple categories of resistance may delay aphid populations from overcoming resistance; therefore, W064 and W068 should be of considerable interest for wheat breeding programmes for sustainable wheat production. However, in Southeast Asia (major wheat producing countries), wheat is also attacked by other aphid species (viz. *R. maidis*, *Sitobion avenae*, *S. miscanthi and S. graminearum*) and resistance to aphids is generally very species specific (Tjallingii, 2006). Thus, future work should focus on detailed comparison of feeding behaviours of different aphid species on Watkins aphid resistant lines to determine the generality and location of aphid resistance. Identification of resistance mechanisms is of great importance, in order to provide effective integrated pest management strategies and possibly informing foresight for resistance management.

## Supporting information


**Figure S1** Morphology of leaf surfaces among different wheat plants. (a) Leaf thickness, (b) size of vascular bundle, (c) size of bundle sheath cell, (d) size of phloem.

**Figure S2** Cell densities in a 100‐μm wide transect section on Triticum aestivum var. Solstice and Watkins landraces W591, W068 and W064.
