# Supplementary figures and images for: Characterisation of bird cherry‐oat aphid (Rhopalosiphum padi L.) behaviour and aphid host preference in relation to partially resistant and susceptible wheat landraces
Source: Ann Appl Biol. 2020 Jul 15;177(2):184–94. doi: 10.1111/aab.12616 (PMC7496520; doi:10.1111/aab.12616)

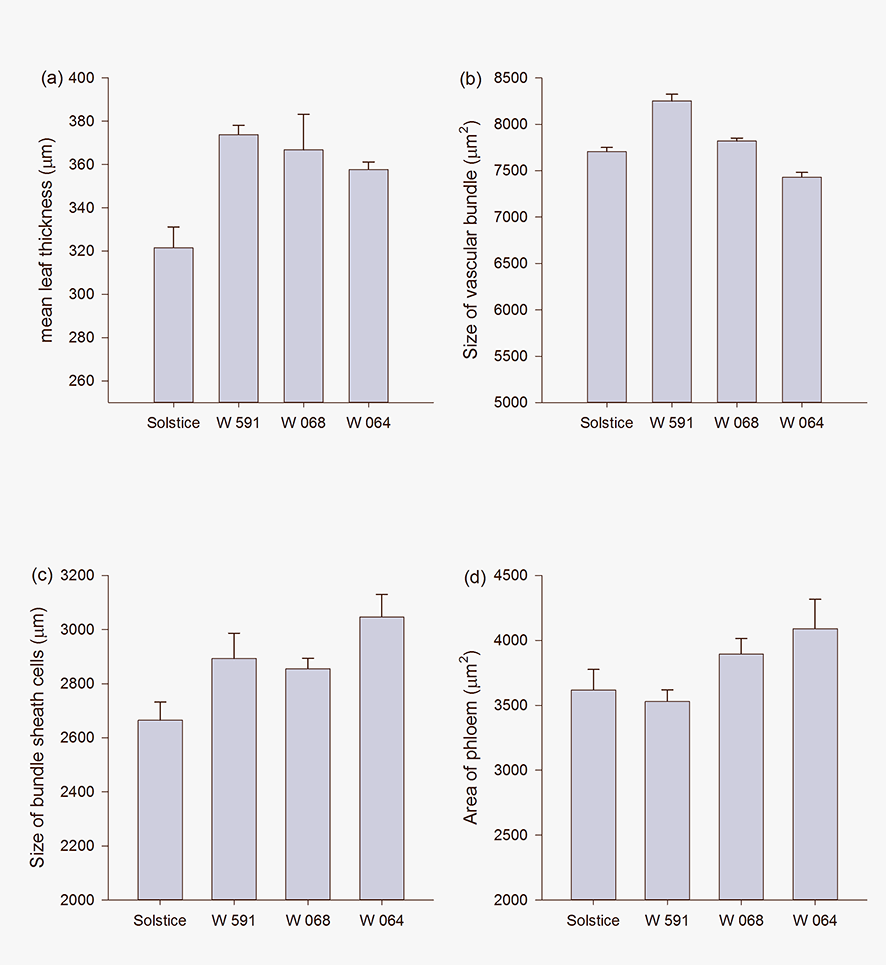

Supplement: Supplementary file 1 — Figure S1 Morphology of leaf surfaces among different wheat plants. (a) Leaf thickness, (b) size of vascular bundle, (c) size of bundle sheath cell, (d) size of phloem. [file AAB-177-184-s001.TIF]

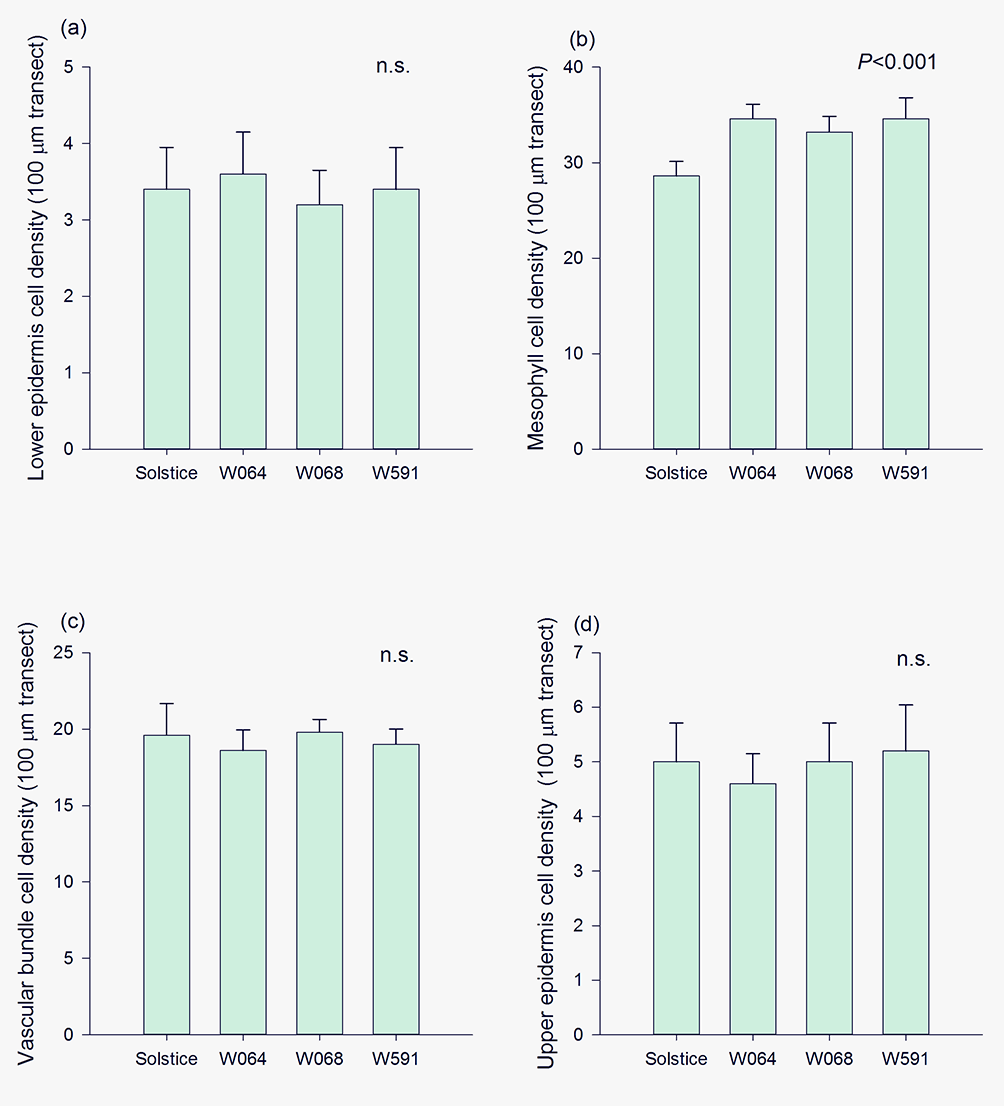

Supplement: Supplementary file 2 — Figure S2 Cell densities in a 100‐μm wide transect section on Triticum aestivum var. Solstice and Watkins landraces W591, W068 and W064. [file AAB-177-184-s002.TIF]
